# Effect of Hydrofluoric Acid, Silane, and Air Abrasion Surface Treatments on the Tensile Bond Strength and Failure Pattern of Glass Fiber Posts: An In Vitro Study

**DOI:** 10.7759/cureus.105510

**Published:** 2026-03-19

**Authors:** Nikhil Sathawane, Rishikesh Meshram, Saurav Bathla, Akanksha Joon, Shaik Rubeena Tabasum, Jay Gohil, Priyanka Razdan, Seema Gupta

**Affiliations:** 1 Department of Conservative Dentistry and Endodontics, Swargiya Dadasaheb Kalmegh Smruti Dental College and Hospital, Nagpur, IND; 2 Department of Conservative Dentistry and Endodontics, Teerthanker Mahaveer Dental College and Research Centre, Teerthanker Mahaveer University, Moradabad, IND; 3 Department of Conservative Dentistry and Endodontics, Faculty of Dental Sciences, Shree Guru Gobind Singh Tricentenary University, Gurugram, IND; 4 Department of Conservative Dentistry and Endodontics, Lenora Institute of Dental Sciences, Rajahmundry, IND; 5 Department of Prosthodontics, Crown and Bridge, K.M. Shah Dental College and Hospital, Sumandeep Vidyapeeth (Deemed to be University), Vadodara, IND; 6 Department of Paediatric and Preventive Dentistry, Yogita Dental College and Hospital, Khed, IND; 7 Department of Orthodontics, Kothiwal Dental College and Research Centre, Moradabad, IND

**Keywords:** air abrasion, bonding, fiber posts, hydrofluoric acid, surface treatment

## Abstract

Introduction: The long-term success of endodontically treated teeth restored with glass fiber posts depends mainly on adequate post-retention. Surface treatment of fiber posts may enhance the bonding between the post and resin cement. The aim of the present study was to evaluate the effect of hydrofluoric acid, a silane coupling agent, and air abrasion with 50 µm alumina particles on the tensile bond strength and failure pattern of glass fiber posts.

Materials and methods: Eighty extracted single-rooted mandibular premolars were endodontically treated and prepared for post-placement. Glass fiber posts were divided into four groups (n = 20): hydrofluoric acid treatment (Group 1), silane application (Group 2), air abrasion with 50 µm alumina particles (Group 3), and distilled water (control as Group 4). The posts were cemented using dual-cure resin cement. After storage, the specimens were subjected to tensile testing using a universal testing machine with a crosshead speed of 3 mm/min. Failure modes were analyzed using a stereomicroscope. Data were analyzed using one-way analysis of variance (ANOVA) and Tukey’s post-hoc test at a significance level of p < 0.05.

Results: The hydrofluoric acid group exhibited the highest mean tensile bond strength (240.12 ± 88.00 MPa), followed by the air abrasion group (209.40 ± 91.15 MPa), silane group (184.13 ± 74.16 MPa), and control group (166.86 ± 72.22 MPa). A one-way ANOVA revealed a statistically significant difference between the groups (p = 0.034). Post-hoc analysis showed a significant difference only between the hydrofluoric acid and control groups. The failure patterns were predominantly mixed, with no significant association between the surface treatment and mode of failure (p = 0.509).

Conclusion: Surface treatment enhanced the retention of glass fiber posts. Hydrofluoric acid and air abrasion improved the bond strength compared to untreated posts; however, hydrofluoric acid showed a significant advantage over the control group. Clinicians should consider both bonding efficacy and potential surface damage when selecting conditioning methods.

## Introduction

Advances in endodontic therapy have significantly improved the long-term retention of teeth that were previously considered nonrestorable. However, endodontically treated teeth are structurally compromised owing to caries, access cavity preparation, canal instrumentation, and loss of internal dentin, making them more susceptible to fracture if not adequately restored [1]. Clinical evidence suggests that the failure of endodontically treated teeth is more often related to inadequate post-endodontic restoration than to endodontic failure itself [2]. Therefore, the restoration of such teeth must aim to re-establish the form, function, and structural integrity while preserving the remaining tooth structure.

When a substantial coronal tooth structure is lost, the use of intraradicular posts is necessary to retain the core and final restoration. Although retentive, traditional metallic posts have been associated with unfavorable stress distribution, increased risk of root fracture, and compromised esthetics owing to metal shadowing [3,4]. By contrast, glass fiber posts have gained popularity because their elastic modulus is closer to that of dentin, resulting in a more favorable stress distribution and reduced incidence of catastrophic root fractures [3]. In addition, their translucency enhances esthetic outcomes and allows effective light transmission for resin cement polymerization.

Despite these advantages, debonding at the post-cement or cement-dentin interface is the most common mode of failure associated with fiber-post restorations. The relatively smooth surface and epoxy resin matrix of prefabricated glass fiber posts limit the micromechanical interlocking with resin cements, thereby compromising retention [5]. To overcome this limitation, various surface treatment methods such as hydrofluoric acid etching, silane application, and air abrasion have been proposed to enhance the surface roughness, chemical bonding, and overall retention of fiber posts [6]. However, the literature presents inconsistent findings regarding the effectiveness of these surface treatments, warranting further investigations.

The aim of the present in vitro study was to evaluate the effects of different surface treatment methods, such as hydrofluoric acid etching, silane application, and air abrasion with alumina particles, on the retention of glass fiber posts. The objectives were to compare the tensile bond strength of surface-treated and untreated glass fiber posts, assess post surface morphology using scanning electron microscopy (SEM), and analyze the mode of bond failure following post dislodgement.

## Materials and methods

This in vitro experimental comparative study was conducted in the Department of Conservative Dentistry and Endodontics at Mahatma Gandhi Vidyamandir's Karmaveer Bhausaheb Hiray Dental College and Hospital, Nashik, India, over a period of approximately six to eight months (October 2022 to March 2023). Ethical approval was obtained from the Institutional Ethics Committee before the commencement of the study (MGV/KBHDC/1033/2021-22). Extracted human teeth were collected following routine dental extractions performed for therapeutic reasons, and written informed consent was obtained from the patients permitting the use of their teeth for research purposes. Patient identifiers were not recorded at any stage.

Inclusion and exclusion criteria

Eighty freshly extracted, caries-free, and fracture-free human single-rooted mandibular premolars were selected for this study. Teeth with cracks, root caries, resorption defects, previous endodontic treatment, calcified canals, and irregular canal morphology were excluded. All teeth were cleaned of calculi and soft tissue remnants using an ultrasonic scaler (Cavitron Bobcat Pro, Dentsply Professional, York, USA). Radiographic evaluation was performed in both buccolingual and mesiodistal directions to confirm the presence of a single canal and to eliminate specimens with anatomical irregularities. The samples were stored in 0.9% normal saline (B. Braun, Mumbai, India) until use to prevent dehydration and preserve the dentinal properties.

The clinical crowns were sectioned 1 mm coronal to the cementoenamel junction using a diamond disc (Summadisk, Shofu Inc., Japan) under water irrigation. The working length was established 0.5 mm short of the apical foramen using a size 10 stainless steel K-file (Mani Inc., Japan). Chemomechanical preparation was performed using ProTaper rotary NiTi files (Dentsply Maillefer, Ballaigues, Switzerland) with an endodontic rotary handpiece (Anthogyr, Sallanches, France) of up to size F3. Irrigation was performed using 3% sodium hypochlorite (Prime Dental Products, Thane, India) and 17% ethylenediaminetetraacetic acid (Dent Wash, Prime Dental Products, Thane, India). Final irrigation was performed with sodium hypochlorite followed by distilled water, and the canals were dried using paper points (ProTaper Universal, Dentsply Maillefer, Switzerland). Obturation was completed using gutta-percha points (ProTaper Universal F3, Dentsply Maillefer, Switzerland) and resin-based sealer AH Plus (Dentsply DeTrey, Konstanz, Germany) using the lateral compaction technique. After 24 hours, post-space preparation was performed using Pezo reamers (Mani Inc., Tochigi, Japan) to a standardized depth of 10 mm.

Glass fiber posts (Reforpost No. 2, 1.3 mm, Angelus, Londrina, Brazil) were randomly allocated into four groups of 20 each according to the surface treatment protocol. In the hydrofluoric acid group (Group 1), the posts were etched with 9.5% hydrofluoric acid (Fisher Scientific, India) for 15 seconds, rinsed thoroughly with water for 30 seconds, and air-dried. In the silane group (Group 2), posts were cleaned in 100% ethyl alcohol (Fisher Scientific, India) and treated with a silane coupling agent (Silano, Angelus, Londrina, Brazil) according to the manufacturer’s instructions, followed by air-drying. In the air abrasion group (Group 3), posts were treated using 50 µm aluminum oxide particles (NOVO, India) in a sandblasting unit (Unident, India) for 10 seconds from a standardized distance of 5 cm and subsequently ultrasonically cleaned in 96% isopropyl alcohol (Fisher Scientific, India). The posts in the control group (Group 4) were immersed in distilled water for 10 seconds and air-dried without additional treatment. Representative specimens from each group were examined under an SEM (S-3400N Type I, Hitachi High-Tech, Tokyo, Japan) at 250x magnification to evaluate the surface topography.

The prepared post spaces were etched with 36% phosphoric acid (Conditioner 36, Dentsply, USA) for 15 seconds, rinsed, and gently dried. A dental adhesive system (Prime & Bond NT, Dentsply Caulk, Milford, USA) along with its self-cure activator, was applied to the canal walls and light-cured using a QHL 75 curing light (Dentsply Caulk, USA). Dual-cure resin cement (Calibra, Dentsply Caulk, Milford, USA) was mixed according to the manufacturer’s instructions and applied to both the post and canal space. The posts were seated under finger pressure and light-cured to ensure complete polymerization. Radiographic confirmation of the post placement was performed.

All specimens were stored in 0.9% normal saline at room temperature for one week prior to testing. Each sample was mounted vertically in an autopolymerizing acrylic resin (DPI RR Cold Cure, Dental Products of India, Mumbai, India) using custom molds, and parallelism was verified using a dental surveyor (Unident, India) to ensure standardized alignment during mechanical testing. Composite core build-up was performed using Filtek Z250 composite resin (3M ESPE Dental Products, St. Paul, MN, USA) in incremental layers and light-cured.

Tensile bond strength testing was carried out using a Universal Testing Machine (STS 248, Star Testing System, India) at a crosshead speed of 3 mm/min until post-dislodgement occurred. The maximum load at failure was recorded in Newtons and converted into megapascals (MPa). The machine was calibrated prior to testing. Following debonding, the specimens were examined under a stereomicroscope (MSZ-TR, Magnus Analytics, India) at 16× magnification to determine the mode of failure as adhesive, cohesive, or mixed.

The maximum load at failure for each specimen was recorded in Newtons (N) during tensile testing using the universal testing machine. Bond strength values were calculated by converting the recorded load into stress (MPa) using the formula: bond strength (MPa) = load at failure (N) divided by the cross-sectional area of the fiber post (mm²). The bonded area was determined based on the circular cross-section of the glass fiber post (diameter 1.3 mm) using the formula A = πr². Because the tensile test measures the dislodgement of the entire post rather than localized interfacial resistance, the calculated values primarily represent normalized load at failure rather than true interfacial stress distribution, which may result in comparatively higher values than those reported in push-out or microtensile bond strength studies.

Sample size estimation

The sample size was estimated using the G*Power software (version 3.1, Heinrich-Heine-Universität, Düsseldorf, Germany). A total of 80 tooth specimens (20 per group) were determined to be sufficient to achieve 95% study power with a 5% alpha error. An effect size of 0.47, derived from a previous study by Schmage et al. [7] that analyzed the tensile strength of fiber-reinforced posts following surface treatment, was used for the calculation.

Statistical analysis

Statistical analysis was performed using the statistical software (SPSS, version 22, IBM Corp., Armonk, NY, USA). Data normality was assessed using the Shapiro-Wilk test. Descriptive data are presented as mean ± standard deviation for tensile strength and frequencies with percentages for failure patterns. One-way analysis of variance (ANOVA) followed by Tukey's post-hoc test was used to compare tensile strength among groups. The chi-square test was used to analyze the associations between groups and failure patterns. Statistical significance was set at p < 0.05.

## Results

Eighty specimens were evaluated, with 20 samples in each group. The mean tensile strength and descriptive statistics are presented in Table 1. The hydrofluoric acid-treated group (Group 1) demonstrated the highest mean tensile bond strength (240.12 ± 88.00 MPa), followed by the air abrasion-treated group (Group 3) (209.40 ± 91.15 MPa), the silane-treated group (Group 2) (184.13 ± 74.16 MPa), and the control group (Group 4), which exhibited the lowest mean value (166.86 ± 72.22 MPa). The 95% confidence intervals showed overlap among groups, particularly between the silane-treated and control groups. Relatively high standard deviation values indicated considerable intragroup variability.

**Table 1:** Descriptive statistics of tensile bond strength (MPa) among study groups. Data expressed as mean ± standard deviation (SD). n = number of specimens per group, MPa = megapascal.

Groups	N	Mean ± SD (MPa)	95% confidence interval for the mean
Hydrofluoric acid (Group 1)	20	240.12 ± 88.00	198.93-281.31
Silane (Group 2)	20	184.13 ± 74.16	149.42-218.84
Air abrasion (50 µm alumina) (Group 3)	20	209.40 ± 91.15	166.74-252.06
Control as distilled water (Group 4)	20	166.86 ± 72.22	133.06-200.67

One-way ANOVA revealed a statistically significant difference in the tensile bond strength among the four groups (p = 0.034), as shown in Table 2. However, the effect size (Cohen’s f = 0.11) indicated a small magnitude of difference, and the residual sum of squares was substantially higher than the between-group sum of squares, confirming the high variability within groups.

**Table 2:** Comparison of tensile bond strength among study groups using one-way analysis of variance (ANOVA). df = degrees of freedom. Significance level set at p < 0.05. *Statistically significant difference, Cohen’s f = measure of effect size (small effect observed).

Source of variation	Sum of squares	df	Mean square	F value	p-value	Effect size (Cohen f)
Between groups	60956.42	3	20318.81	3.04	0.034*	0.11
Within groups (residual)	508604.81	76	6692.17			

Post-hoc analysis using Tukey’s test (Table 3) demonstrated a statistically significant difference only between the hydrofluoric acid-treated group (Group 1) and the control group (Group 4) (mean difference = 73.26 MPa, p = 0.030; 95% CI: 5.30-141.21). All other pairwise comparisons were not statistically significant (p > 0.05). Thus, the overall ANOVA significance was primarily attributable to the difference between the hydrofluoric acid-treated and control groups.

**Table 3:** Post-hoc comparison of tensile bond strength using Tukey’s test. CI = confidence interval. Significance level set at p < 0.05. *Statistically significant difference.

Pairwise groups	Mean difference (MPa)	t value	p-value	95% CI (lower limit)	95% CI (upper limit)
Hydrofluoric acid (Group 1) vs. Silane (Group 2)	55.99	2.16	0.143	-11.96	123.94
Hydrofluoric acid (Group 1) vs. Air abrasion (Group 3)	30.72	1.19	0.637	-37.24	98.67
Hydrofluoric acid (Group 1) vs. Control (Group 4)	73.26	2.83	0.030*	5.30	141.21
Silane (Group 2) vs. Air abrasion (Group 3)	-25.27	-0.98	0.763	-42.68	93.22
Silane (Group 2) vs. Control (Group 4)	17.27	0.67	0.909	-50.69	85.22
Air abrasion (Group 3) vs. Control (Group 4)	42.54	1.64	0.360	-25.41	110.49

The distributions of the failure modes are listed in Table 4. Mixed failure was the predominant mode in all the groups. In the hydrofluoric acid-treated group, 70% of the specimens exhibited mixed failure, with equal distribution between 0 and 50% and 50-100% resin cement coverage. The silane-treated, air-abrasion-treated, and control groups also predominantly demonstrated mixed failures, most commonly in the 0-50% resin cement coverage category (50-60%). Adhesive failure between the post and resin cement was the least frequent in the air abrasion-treated group (5%). Chi-square analysis revealed no statistically significant association between surface treatment and failure pattern (χ² = 5.26, p = 0.509), indicating a comparable failure behavior among all groups.

**Table 4:** Distribution of failure modes among study groups (Chi-square test). n = number of specimens. Percentages calculated within each group (n = 20). No statistically significant association between surface treatment and failure pattern (p > 0.05).

Groups	Adhesive failure between post and resin cement n (%)	Mixed failure (0–50% cement coverage) n (%)	Mixed failure (50–100% cement coverage) n (%)	Chi stats	p- value
Hydrofluoric acid (Group 1)	6 (30%)	7 (35%)	7 (35%)	5.26	0.509
Silane (Group 2)	4 (20%)	10 (50%)	6 (30%)		
Air abrasion (Group 3)	1 (5%)	12 (60%)	7 (35%)		
Control as distilled water (Group 4)	3(15%)	11 (55%)	6 (30%)		

**Figure 1:**
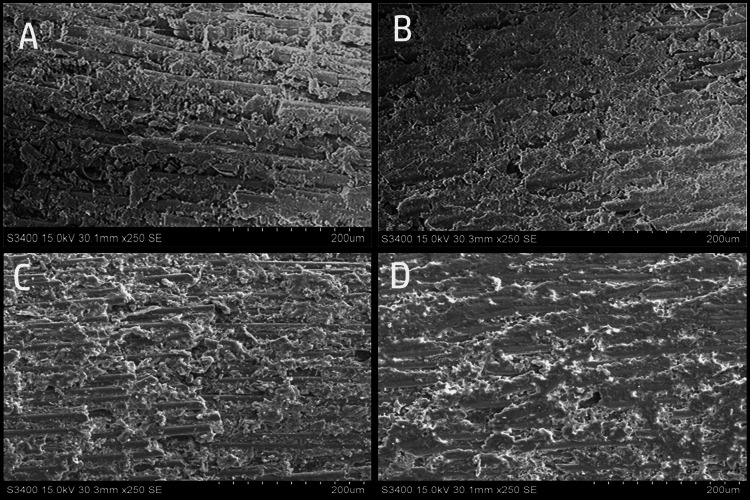
Scanning electron microscopic (SEM) images (250x magnification) of glass fiber post surfaces following different surface treatments: (A) hydrofluoric acid-treated group showing exposed fibers and pronounced surface irregularities, (B) silane-treated group showing relatively smooth surface with limited matrix alteration, (C) air abrasion (50 µm alumina)-treated group showing increased surface roughness and partial fiber exposure, (D) control group (distilled water) showing smooth surface with intact resin matrix and parallel-oriented fibers. Scale bar = 200 µm. These are the original SEM images of samples from the study.

SEM evaluation revealed distinct surface alterations among the different surface treatment groups (Figure 1). The hydrofluoric acid-treated group (Figure 1A) demonstrated pronounced surface irregularities with evident dissolution of the epoxy resin matrix and clear exposure of the glass fibers. The fibers appeared fractured and unevenly distributed, indicating aggressive surface modification. The silane-treated group (Figure 1B) showed relatively minimal surface changes with a comparatively smoother appearance and limited disruption of the resin matrix. The glass fibers were visible but less exposed, suggesting that silane application alone did not significantly alter the surface topography. The air abrasion (50 µm alumina)-treated group (Figure 1C) exhibited increased surface roughness with partial interruption of fibers and removal of the superficial resin matrix. The surface appeared more irregular than that of the silane group but less aggressive than that of the hydrofluoric acid group. The control group, distilled water (Figure 1D), displayed a relatively smooth and uniform surface with parallel-oriented fibers embedded within an intact resin matrix and minimal surface irregularities.

## Discussion

The present in vitro study evaluated the effects of different surface treatments on the tensile bond strength and failure pattern of glass fiber posts. The results demonstrated that the hydrofluoric acid-treated group exhibited the highest mean tensile bond strength, followed by the air abrasion, silane, and control groups. Although one-way ANOVA showed a statistically significant difference among the groups (p = 0.034), post-hoc analysis revealed that the significant difference was primarily between the hydrofluoric acid group and the control group.

The superior performance of the hydrofluoric acid treatment may be attributed to its ability to selectively dissolve the epoxy resin matrix and expose the underlying glass fibers. This creates a roughened, micro-retentive surface that enhances micromechanical interlocking with resin cement. The SEM observations in the present study confirmed the surface irregularities and exposure of the fibers in the hydrofluoric acid group. Similar findings were reported in a previous study [8], which observed increased surface roughness and micro-retentive spaces after hydrofluoric acid conditioning of fiber posts. However, because of its caustic nature, hydrofluoric acid may cause aggressive surface changes, including damage to the resin matrix and glass fibers, potentially compromising the mechanical properties of the post. Variations in acid concentration and application time across studies further contribute to inconsistent outcomes. Therefore, although hydrofluoric acid can improve bond strength, its use should be approached cautiously, and less aggressive surface treatments may be preferable in clinical practice to avoid structural weakening of the post [9-11].

Air abrasion with alumina particles also improved the bond strength compared to the control group, although the difference was not statistically significant when compared to hydrofluoric acid. Airborne particle abrasion increases the surface roughness by mechanically abrading the resin matrix and partially exposing the fibers. The SEM analysis in this study demonstrated the interruption of fibers and increased surface irregularities in the air-abrasion group. These findings are consistent with those of Sahafi et al. [11], Karunakaran et al. [12], and Machry et al. [13], who concluded that sandblasting significantly improves the retention of glass fiber posts. The comparable performance of hydrofluoric acid and air abrasion in the present study suggests that both chemical and mechanical surface treatments can effectively enhance micromechanical retention.

The silane-treated group demonstrated moderate improvement compared to the control group, but did not significantly differ from either the hydrofluoric acid or air abrasion groups. Silane acts as a chemical coupling agent, forming a siloxane bond between the inorganic glass fibers and the organic resin matrix. However, its effectiveness depends on adequate exposure to glass fibers. If the epoxy resin matrix remains largely intact, silane application alone may not substantially enhance the bonding. Similar observations were made by Choi et al. [14], who reported no significant improvement with silanization alone compared with the control. Perdigao et al. [15] and Campelo et al. [16] also suggested that post-surface pretreatment with silane may not significantly influence bond strength. According to a systematic review by Souza et al. [17], combining chemical and physical methods can significantly improve the bond strength between glass-fiber-reinforced composite posts and resin-matrix cements.

The control group treated with distilled water only showed the lowest bond strength values. This finding supports the concept that untreated fiber posts have relatively smooth surfaces with a limited potential for micromechanical interlocking. Previous investigations have consistently reported lower bond strengths in non-treated posts owing to inadequate surface roughness and weak interfacial bonding [7,8].

Failure mode analysis revealed predominantly mixed failures across all groups, with no statistically significant association between surface treatment and failure patterns. The predominance of mixed failures suggests that bonding occurred at both the post-cement and cement-dentin interfaces. Adhesive failure between the post and cement was the least common in the air-abrasion group, which may indicate improved interfacial integrity. These findings agree with those of Albashaireh et al. [18], who reported predominantly mixed failures following airborne particle abrasion.

Although hydrofluoric acid demonstrated the highest mean tensile bond strength, the effect size was small (Cohen’s f = 0.11), indicating that the practical magnitude of the difference among groups was limited. The relatively high standard deviations observed in all groups reflect biological variability among extracted teeth and differences in dentin substrate, which may have influenced the bond strength outcomes.

Although the hydrofluoric acid and air abrasion groups demonstrated higher mean tensile bond strength values compared with the control group, considerable intra-group variability was observed, as reflected by the relatively high standard deviation values. Additionally, the calculated effect size (Cohen’s f = 0.11) indicated a small magnitude of difference among groups, and post-hoc analysis showed a statistically significant difference only between the hydrofluoric acid and control groups. Therefore, while surface treatments appeared to improve mean retention values, the magnitude of improvement should be interpreted cautiously. The findings suggest a potential beneficial effect of certain surface treatments rather than a definitive enhancement of retention.

Clinical implications

From a clinical perspective, surface treatment of glass fiber posts appears to enhance retention compared with no treatment. Hydrofluoric acid conditioning and air abrasion both improved tensile bond strength, suggesting that clinicians may consider these procedures when aiming to optimize post-retention. However, the technical sensitivity and safety concerns associated with intraoral hydrofluoric acid application must be considered; in the present study, the acid treatment was performed extra-orally on the fiber posts prior to insertion. Air abrasion may represent a more practical and safer alternative in clinical settings. Silane application alone may not be sufficient unless combined with a surface roughening method that exposes the glass fibers. Improved post retention may reduce the risk of post dislodgement, which is one of the most common modes of failure in endodontically treated teeth restored with fiber posts.

Limitations

This study was conducted under in vitro conditions, which do not fully replicate the complex oral environment. Factors, such as thermal cycling, occlusal loading, moisture contamination, and aging, were not simulated. Although standardized, the sample size may still contribute to variability. In addition, only the tensile bond strength was evaluated; other tests, such as push-out bond strength or long-term fatigue testing, may provide further insight. Future studies incorporating thermomechanical aging and clinical trials are necessary to validate these findings and determine their long-term clinical performance.

## Conclusions

Within the limitations of this in vitro study, surface modification of glass fiber posts appears to influence their interaction with resin cement and may contribute to improved interfacial integrity. Alterations in surface topography and fiber exposure play an important role in enhancing micromechanical and chemical bonding mechanisms. However, the overall improvement observed with surface conditioning protocols was modest, suggesting that retention of fiber posts is multifactorial and not solely dependent on post surface treatment. While certain conditioning methods produced greater surface alterations, aggressive approaches may also compromise structural integrity and require careful clinical judgment. Therefore, the selection of a surface treatment strategy should balance potential bonding enhancement with preservation of post properties and procedural safety. Further long-term and clinically simulated studies are required to determine the practical relevance of these surface modification techniques in routine restorative practice.
